# Intimate partner physical violence and associated factors in reproductive age married women in Aksum Town, Tigray, Ethiopia 2018, and community based study

**DOI:** 10.1186/s13104-019-4615-3

**Published:** 2019-09-24

**Authors:** Alem Girmay, Teklewoini Mariye, Degena Bahrey, Berihu Hailu, Assefa Iyasu, G/amlak G/medhin, Binyam Demisse, Girmay Teklay

**Affiliations:** 1 Department of Adult Health Nursing Specialty, School of Nursing, College of Health Science and Comprehensive Referral Hospital, Aksum University, Aksum, Ethiopia; 2 Department of Pediatrics and Child Health Specialty, School of Nursing, College of Health Science and Comprehensive Referral Hospital, Aksum University, Aksum, Ethiopia

**Keywords:** Physical violence, Married Women, Aksum town, Ethiopia

## Abstract

**Objective:**

As reports indicated about 1 in 3 of women worldwide have experienced physical violence but there is no enough reports on the current status of the act in Aksum town so this study intended to identify the prevalence and factors associated with physical violence of reproductive age married women in Aksum town Tigray Ethiopia.

**Result:**

A total of 398 women were enrolled in the study and making a response rate of 100%. 112 (28.1%) women had physical violence in their lifetime. Educational level of women (AOR = 2.2; 95% CI 1.28, 6.7), Occupation of women’s (AOR = 3.8; 95% CI 2.32, 12.8), age of husband (AOR = 5.2; 95% CI 2.3, 11.5), husbands having other wife (AOR = 7.8; 95% CI 4.2, 18.9) and husbands having alcohol habits (AOR = 3.8; 95% CI 1.74, 14.7) had significant association with physical violence.

## Introduction

According to the definition of the world health organization violence is the intentional use of physical force or power against another person that results in injury, death, psychological harm, mall development or deprivation and physical violation is a type of violence where someone is bodily suffered/harmed due to physical force [1–4].

Physical violence against women is increasingly recognized by the international community as it is a significant violation of human rights, and so many studies done in response to this in different regions, countries, cultures and socioeconomic classes, and the studies indicating women’s in developing countries experience higher rate of intimate partner physical violence than women’s from developed countries [5].

Physical violence affects women’s physical and mental health directly like injury and indirectly like chronic health problems that can develop from prolonged stress. As severe as physical violence exists there will be a greater impact on the women’s health and the health impact seem cumulative in a long duration [5, 6].

Intimate physical violence can have huge personal, social and economic effects on women’s status; it may result in conflict or dissatisfaction in the relationship of the partners, male partner dominance in the family, economic instability and high levels of general violence in society [7].

Data availability on physical violence is high in Africa, according to available data on the prevalence of physical violence, indicating that the highest prevalence was in Africa, in almost countries lifetime prevalence reported over 40% in the range varied from 14% in Comoros and 64% in Democratic republic Congo but in Asian countries it ranges from 13% in Azerbaijan to 40% in Timor-Leste. In Tennessee, 40% of women had intimate partner physical violence in their lifetimes. As current reports even the prevalence of physical violence declines it is still high in Ethiopia, which is 49% [8–11].

As reports in EDHS, 2016 physical violence is higher among formerly married women, which is 45%, among age groups of 40–49 years old women’s which is 38%, those living in rural areas (36%), and women in Oromo (39%), Harare (38%), Amara (37%) and in Tigray (25.5%). Physical violence decreases with increasing educational level and household wealth [12, 13].

According to the WHO estimation about 1 in 3 of women worldwide have experienced physical violence which was reported as a pandemic problem. Globally, physical violence against women, recognized as a fundamental human rights violation, is widely prevalent across high, middle and low-income countries, but still the health system not adequately addressing the problem of intimate partner physical violence and for this reason it is contributing to comprehensive multi-sectoral problems/responses [1, 3, 5, 8, 10, 14].

As reports in Ethiopia around 23% of women have ever experienced intimate partner physical violence since 15 years old, and the most common perpetrator of physical violence among ever-married women were current husband/partner which covers around 68%. Though there are many studies in Ethiopia there is no study in Aksum, and IPV is routine in the outpatient visit in the area, as well as we absorb the problem in our daily activities, that is why we are intended to study focusing on intimate partner physical violence [13, 15].

## Main text

### Study area and period

The study was conducted in Aksum town which is located in the Central Zone of Tigray Regional State, at a distance of 1024 km from Addis Ababa. The total population of Aksum town is 60,766, with 30,991 (51.0%) females and 29,775 (49.0%) males. Administratively the town is divided into five Keble [16].

#### Study designs and population

Population based cross sectional study was conducted to study reproductive aged married/cohabited women living in Aksum selected three Keble’s during the study period.

#### Sampling size and sampling technique and procedure

The sample size was determined using single population proportion with assumptions, prevalence = 20.6% [17], 95% CI, 5% marginal error, correction formula and 10% non-response rate, yielding to final sample size of 398, it also assumed sample size calculation for different associated factors. To select study participants at their permanent place of residence, systematic sampling technique was used.

#### Data collection tool, procedure and data quality control

Data was collected using a pretested semi structured questionnaire to assess women Socio demographic characteristics, women’s husband characteristics and the women’s experience of IPV. The questionnaire was originally prepared in the English language, and then translated to the local language. Data were collected in a timeframe of 4 months by trained data collectors and three supervisors were supervised the data collection. Training was given for data collectors and supervisors. Supervisors and researchers were strictly supervised data collection. Data were cleaned before commencement of the analysis. Finally for victims of IPV “need based” support were given in their interested time and place like hospital, church or local organization [18].

### Operational definitions

#### Intimate partner physical violence

Self-reported one or more episodes of physical threats in which husband/partner battered his wife either by push, shake, or throw something at; slap; twist arm or punch you with his/her fist or with something that could hurt; kick, drag, or beat up; or threaten or attack with a knife, gun, or any other weapon that can result in physical harm of married women [1, 19].

### Data analysis

Data were entered to EPI data version 3.02, transported and analyzed using SPSS version 20.0. Both measures of central tendency and dispersion were calculated. Bivariate logistic regression was run to infer an association between dependent and independent variables and independent variables with a p-value 0.25 were inserted to multivariable logistic regression to obtain significant variables associated with the dependent variable. The significance level was declared at a p-value 0.05.

#### Ethical considerations

This study was reviewed and approved by the Research Committee, Health Science College and Comprehensive Specialized Hospital of Aksum University. Data were collected after full written informed consent was obtained from each study subjects. Consent and assent also obtained from their parents for those whose age was less than 16 years old.

### Results

#### Socio demographic characteristics of participants

A total of 398 women enrolled in the study and yielding a response rate of 100%. Around 175 (44%) of the participants were in the age group ≥ 31 years old and 280 (70.4%) married at the age of 16–20 years old. Around 56.8.2% of the participants were orthodox by their religion. Regarding the occupational status of the participants, 245 (61.6%) were housewife. 198 (49.7%) was 2–4 parity and 95 (23.9%) women were pregnant during the study period (Table 1).

**Table 1:** Socio demographic and socioeconomic characteristics of married reproductive age women, Aksum town, 2018 (n = 398)

Variable	Frequency	Percent
Age of women’s (years)		
< 15	12	3.0
16–20	64	16.1
21–25	75	18.0
26–30	72	18.1
≥ 31	175	44.0
Age at marriage		
< 15 years	34	8.5
16–20	280	70.4
21–25	75	18.8
≥ 26	9	2.3
Religion		
Muslim	155	33.8
Protestant	15	3.8
Orthodox	226	56.8
Catholic	2	0.5
Educational level of women’s		
Have no formal education	60	15.1
Able read and write	17	4.3
Elementary 1–4 grade	37	9.3
Elementary 5–8 grade	98	24.6
High school 9–12	116	29.1
College and above	70	17.6
Occupation of women’s		
Housewife	245	61.6
Governmental employment	66	16.6
Daily laborer	21	5.3
Private employee	32	8
Merchant	28	7.5
Women’s association leader	6	1.1
Parity		
1	91	22.9
2–4	198	49.7
5–7	71	17.8
≥ 8	7	1.8
Family size		
< 2	44	11.1
3–5	214	53.8
6–8	105	26.4
> 9	26	6.5
Women’s pregnant currently		
Yes	95	23.9
No	303	76.1
Age of pregnancy in months		
≤ 3	10	2.5
4–7	35	8.8
> 7	3	0.8
Women’s having habits		
Yes	78	19.6
No	320	80.4
How many years do you stayed with your current husband/parent		
< 1	300	75.4
2–5	26	6.5
6–10	23	5.8
16–20	7	1.8
Your current husband is your husband/parent of		
First	348	87.4
Second	50	12.6
Age of husband/parent		
< 25	6	1.5
26–30	70	17.6
31–35	56	14.1
36–40	91	22.9
41–45	44	11.1
> 46	121	30.4
Husband/parent educational status		
Have no formal education	24	6.0
Able to read and write	26	6.5
Elementary 1–8 grade	33	8.3
High school 9–12	103	25.9
College and above	212	53.3
Husband/parent occupation		
Governmental employment	184	46.2
Daily laborer	97	24.4
Private employee	66	16.6
Merchant	43	10.8
NGO	8	2
Husband/parent have other wife		
Yes	55	13.8
No	343	86.2
How many wives does have your husband out of you		
1	38	9.5
≥ 2	17	4.3
What type of habit does have your husband/parent		
Alcohol	100	25.1
Cigarette	15	3.8
Khat	19	4.8
Which wife of your current husband/parent you are		
First	341	85.7
Second	50	12.6
Third	2	0.5
How was your marriage initiated		
Friend	44	11.1
By yourself	40	10.1
Relative	307	77.1
Neighbors	7	1.8
Your family’s status by the community		
Highly respected	272	67.5
Moderately respected	113	29.3
Less respected	13	3.2
Economical back ground of your family		
High class	98	24.6
Moderate class	283	71.1
Lower class	17	4.3

#### Intimate partner physical violence

Out of the 398 study participants, 112 (28.1%) and 27 (6.8%) married reproductive age women had intimate partner physical violence in their lifetime and in the last 3 months respectively, from the physically violence reproductive age women, 88 (22.1%) had conflict with their husband, 35 (8.8%) and 65 (16.3%) battered by their husband usually and sometimes respectively. A total of 48 (8.7%) and 27 (6.8%) respectively had conflict and battered in the last 3 month (Table 2).

**Table 2:** Types of physical violence in married reproductive age women in their lifetime and in the last 3 months, Aksum town, 2018 (n = 398)

	Frequency	Percent
Do you support that a women to be battered by her husband whether she is right or wrong		
Yes	79	22.9
No	265	76.8
Have you seen a conflict between you and your husband, since your marriage		
Yes	88	22.1
No	310	77.9
How frequent was the conflict between you and your husband, since your marriage		
Usually	13	3.3
3 times in a week	2	0.5
1 times in a week	3	0.8
Sometimes	29	7.3
Rarely	40	10.1
What was the cause of a conflict between you and your husband, since your marriage		
Not wanted marriage	2	0.5
Alcoholic husband	16	4.0
Husband has other wife	14	3.5
Economical problem	12	3.0
You don’t obey your husband properly	3	0.8
Initiated by relatives	9	2.3
Husbands bad habit (cigarette, Khat)	1	0.3
Initiated by neighbors	3	0.8
Does your husband battered you, since your marriage		
Yes	112	28.1
No	386	71.9
How frequent was your husband battered you, since your marriage		
Usually (daily to 2×/week)	35	8.8
Sometimes (1×/day to 1×/3 month)	65	16.3
One time only	12	3.0
Have you got any injury by your husband		
Yes	92	23.1
No	20	5.02
What was the outcome injury by your husband		
Small laceration or scare	48	12
Swelling on the face/other area	24	6.0
Abortions	5	1.25
Simple puncture	13	3.3
Simple laceration	2	0.5
Have you seen a conflict between you and your husband for the last 3 months		
Yes	48	8.7
No	350	91.3
Has your husband battered you for the last 3 months		
Yes	27	6.8
No	85	21.4
How frequent was battered you for the last 3 months		
One times	16	4.0
Two times	11	2.8
Have you got any injury battered you		
Yes	12	3.01
No	15	3.8
What was the outcome of battered by husband for the last 3 months		
Small laceration or scare	7	1.8
Swelling on the face/other area	2	0.5
Fractures and dislocations	2	0.5
Abortions	1	0.25
What type of weapon used for physical violence during the last 3 months		
Slapped or hit with fist	19	4.8
Kicked or hit with leg	3	0.8
Slashed with thin stick	4	1.0
Hit or beaten with stick/iron bar	1	0.25
Due to the injury have you visit nearby health institution		
Yes	5	1.3
No	7	1.8
Have you ever been separated due to the conflict		
Yes	17	4.9
No	327	94.8

#### Factors associated with intimate partner physical violence

After controlling the confounding effect educational level of women, occupation of women’s, age of women’s husband, women’s having husbands with other extra wife and women’s having husbands with alcohol/smoking habits had significant association with intimate partner physical violence in the multivariate logistic regression model (Table 3).

**Table 3:** Factors associated with physical violence of married reproductive age women, Aksum town, 2018 (n = 398)

Dependent variables	Physical violence		COR 95% CI	AOR 95% CI
	Yes	No		
Educational level of women				
Have no formal education**	16	44	0.37 (0.11, 0.93)	2.2 (1.28, 6.7)
Able read and write	2	15	1.88 (1.06, 3.47)	4.87 (2.44, 7.89)
Elementary 1–4 grade	15	22	0.94 (0.12, 0.99)	3.7 (2.01, 7.6)
Elementary 5–8 grade	25	73	1.002 (1.0001, 2.7)	4.6 (1.2, 17.9)
High school 9–12**	31	85	1.35 (1.1, 4.9)	0.34 (0.11, 0.78)
College and above	23	47	1	1
Occupation of women’s				
Housewife**	68	177	1.47 (1.23, 5.6)	3.8 (2.32, 12.8)
Governmental employment	18	48	1.5 (1.3, 7.9)	0.22(0.12, 0.56)
Daily laborer**	3	18	3.4 (1.9, 5.8)	4.2 (3.11, 8.22)
Private employee	10	20	2 (1.6, 8.9)	0.37 (0.23, 0.77)
Women’s association leader	13	23	1	1
Age of women’s husband (years)				
≤ 30**	23	53	0.76 (0.11, 0.94)	5.2 (2.3, 11.5)
31–40	41	106	0.85 (0.23, 0.89)	2.1 (1.76, 3.7)
≥ 41	41	124	1	1
Husbands having other wife				
Yes**	20	35	0.64 (0.31, 0.95)	7.8 (4.2, 18.9)
No	93	251	1	1
Husbands with alcohol/smoking habits				
Yes**	52	60	3.8 (2.3, 13.7)	3.8 (1.74, 14.7)
No	204	62	1	1

**Variable categories having p-value < 0.05

### Discussions

In Ethiopia and other developing countries as well as in the study area, intimate partner physical violence is a major health problem, but in the specified study area there was no enough evidence related to intimate partner physical violence, hence we intended to show the status and its associated factors of it. Based on this, our study result showed that out of the total; 28.1% and 6.8% had reported lifetime and in the last 3 months respectively. Our study finding is also lower than comparable to a study conducted in Shanghai, China (31.9%) [19], Kusheshwor, Sindhuli, Nepal (29.6%) [20]. Another study conducted in Uganda (41%), Nigeria 30.5%, both study findings were higher as compared to our study finding [21, 22]. Our Study finding also less than from the study finding in Ethiopia from 2000 to 2014 which ranged from 31 to 76.5% [4]. Our Study finding also less than from the study conducted in Debre Tabor town in 2015, which showed, 56.1% lifetime and 27.9% in the last 12 months [23]. Additionally, another Study conducted in Shimelba refugee camp was 25.5% [12], and Southwest Ethiopia, 64.7% [24], Hawzen, 38.6% which is higher than our study finding [25].

In this study Women’s educational status had significantly associated with intimate partner physical violence among those reproductive aged, married women’s. Women’s who had no formal education was 2 times more likely to experience intimate partner physical violence than college and above educational level women’s, and those grade 9–12 were 0.34 times less likely to report intimate partner physical violence. This is consistent with earlier studies finding of WHO, 2010 [6], Shire Endaslassie, Ethiopia [17], Iran [26], Nepal [20] and in contrast to the study finding in Mozambique [27], the possible reason for this difference might be due to study area and/or study participants Socio-demographic characteristics difference.

Being a housewife in their occupation was 4 times more likely to experience intimate partner physical violence than women’s in women’s association leader occupation. Since housewife’s are most of the time economically dependent on their husband, they are more vulnerable to intimate partner physical violence and this is consistent with studies finding in Nigeria [22], Nepal [20], Pakistan [28], and in contrast with study finding in Southwest Ethiopia, this could be as women’s exposure to the larger society, husband/partner might be violent on the working women just to prevent women from working outside the home as a way of controlling them and involvement of women in economic activity might be considered as a challenge in power sharing with man in the home [24].

Women’s being daily laborer in their occupation was 4 times more likely to experience intimate partner physical violence than women’s in women’s association leader occupation. This was in line with study findings in Shanghai, China [19], Nepal [20] and Pakistan [28].

A woman who had husbands in the age group ≤ 30 years old was 5 times more likely to report intimate partner physical violence than those who had husbands ≥ 41 years old. This result is in contrast with study finding in Hawzen, Ethiopia [25], in our finding as age of husband increasing experience of intimate partner physical violence decreases irrespective of women’s age, but in the study in Hawzen as women’s age increases the experience of intimate partner physical violence increases irrespective of husbands age, it was also in contrast with the report in WHO [14] and Iran [26].

Women’s where their husband had another extra wife were 8 times more likely to report intimate partner physical violence than those Women’s where their husband had no extra wife. This is in line with studies result in WHO, 2010 [6], Shimelba [12], Uganda [29] and Northwest Ethiopia [23].

Participants who had husbands with habit (alcohol/smoking) were 4 times more likely to report Women’s where their husband physical violence than women’s with non-alcoholic/smoker husband. This was consistent with the studies finding in WHO, 2010 [6], WHO, 2013 [10], Northwest Ethiopia [23], Gondar [30], Shimelba, Ethiopia [12], Arsi [31] and Iran [26], Nepal [20], Uganda [21] and Shire Endaslassie, Ethiopia [17].

### Conclusions

A significant number of married reproductive age women’s had experienced intimate partner physical violence. Being women with have no formal education, house wife, having husband ≤ 31 years old, women’s having husband with other additional wife and having an alcoholic/smoker husband were independent variables significantly associated with intimate partner physical violence.

## Limitations

The result depends only on the response of participants, so that there might be chance of recall bias. Since we only focus on the physical violence, this may make under or over reporting. During participants’ selection, our study had not included homeless and street married women. Interviewer and respondent power differential may be happened but we tried to minimize it using location, location, location (interview in private, participants choosing, where she feels comfort) and rapport.

## Data Availability

The datasets used and/or analyzed during the current study are available from the corresponding author on reasonable request.

## Abbreviations

CI: confidence interval
AOR: adjusted odd ratio
SPSS: Statistical Package for Social Sciences
